# Supplementary figures and images for: Isolation of Lineage Specific Nuclei Based on Distinct Endoreduplication Levels and Tissue-Specific Markers to Study Chromatin Accessibility Landscapes
Source: Plants (Basel). 2020 Nov 3;9(11):1478. doi: 10.3390/plants9111478 (PMC7692515; doi:10.3390/plants9111478)

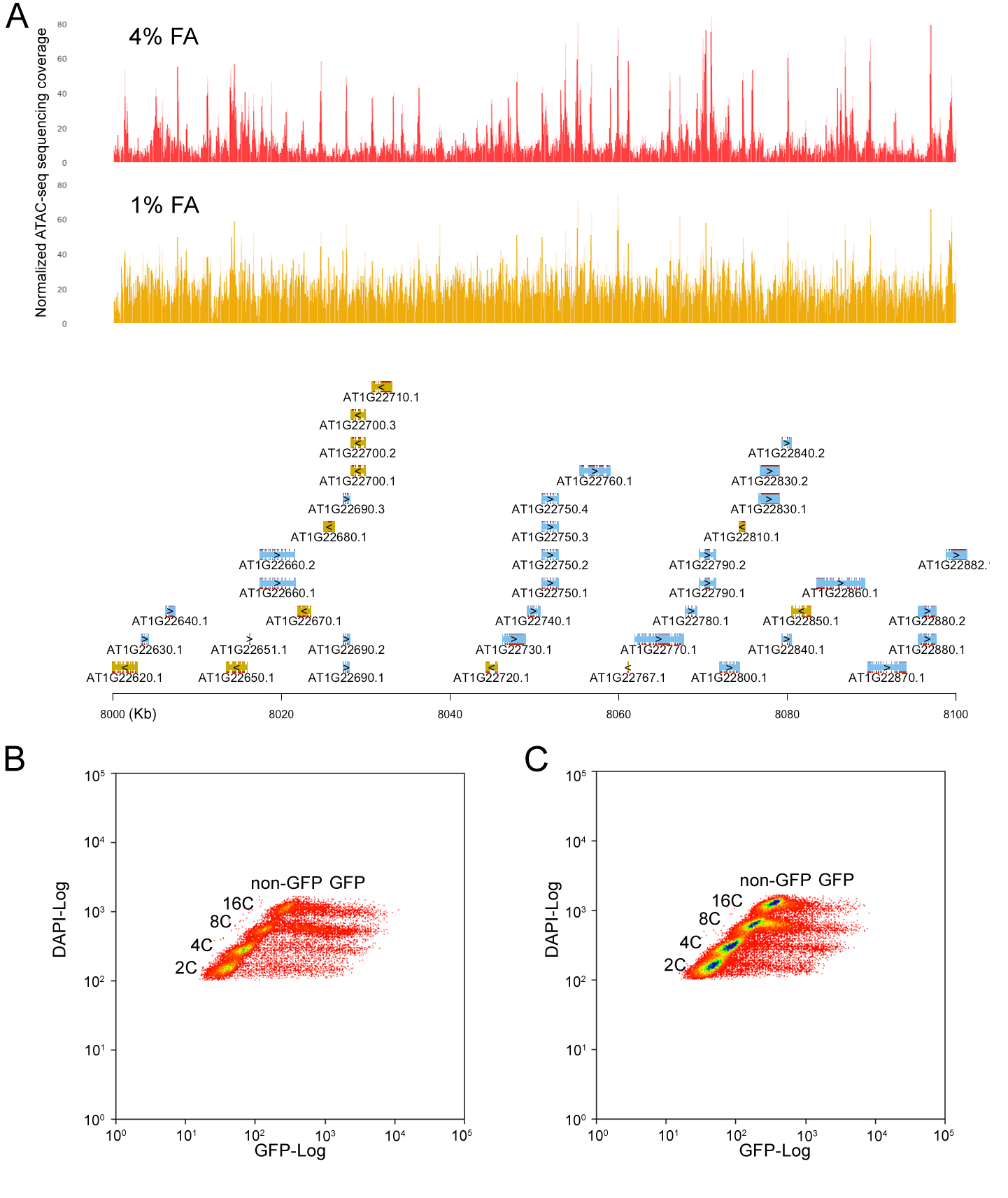

Supplement: Supplementary file 1 [file plants-09-01478-s001.zip › FigS1_v6.tif]

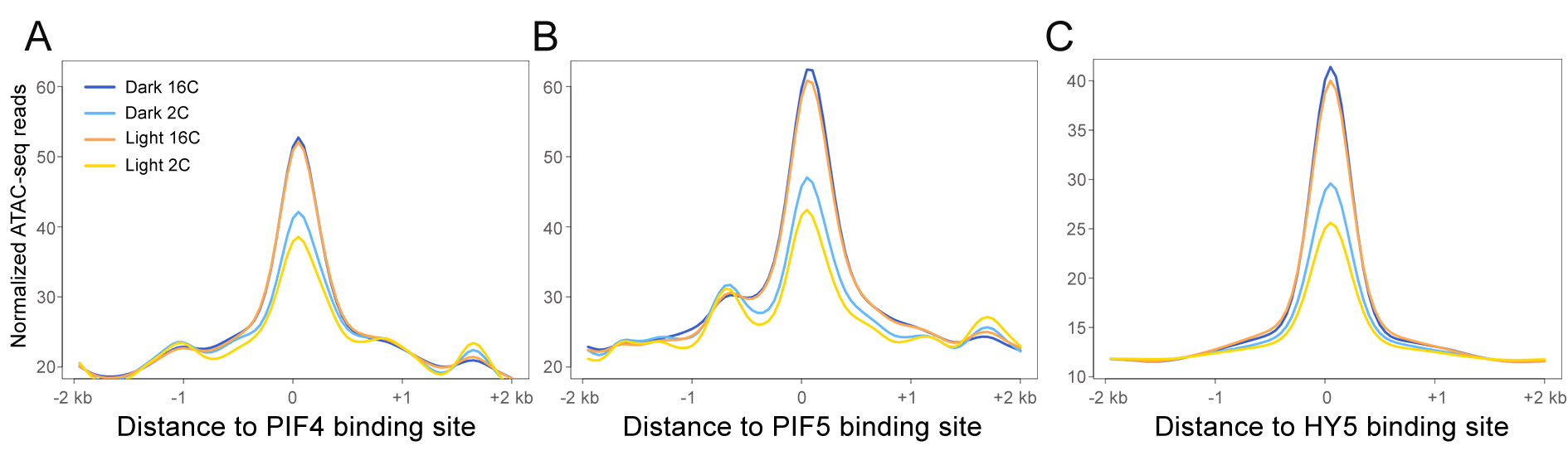

Supplement: Supplementary file 1 [file plants-09-01478-s001.zip › FigS2_v6.tif]

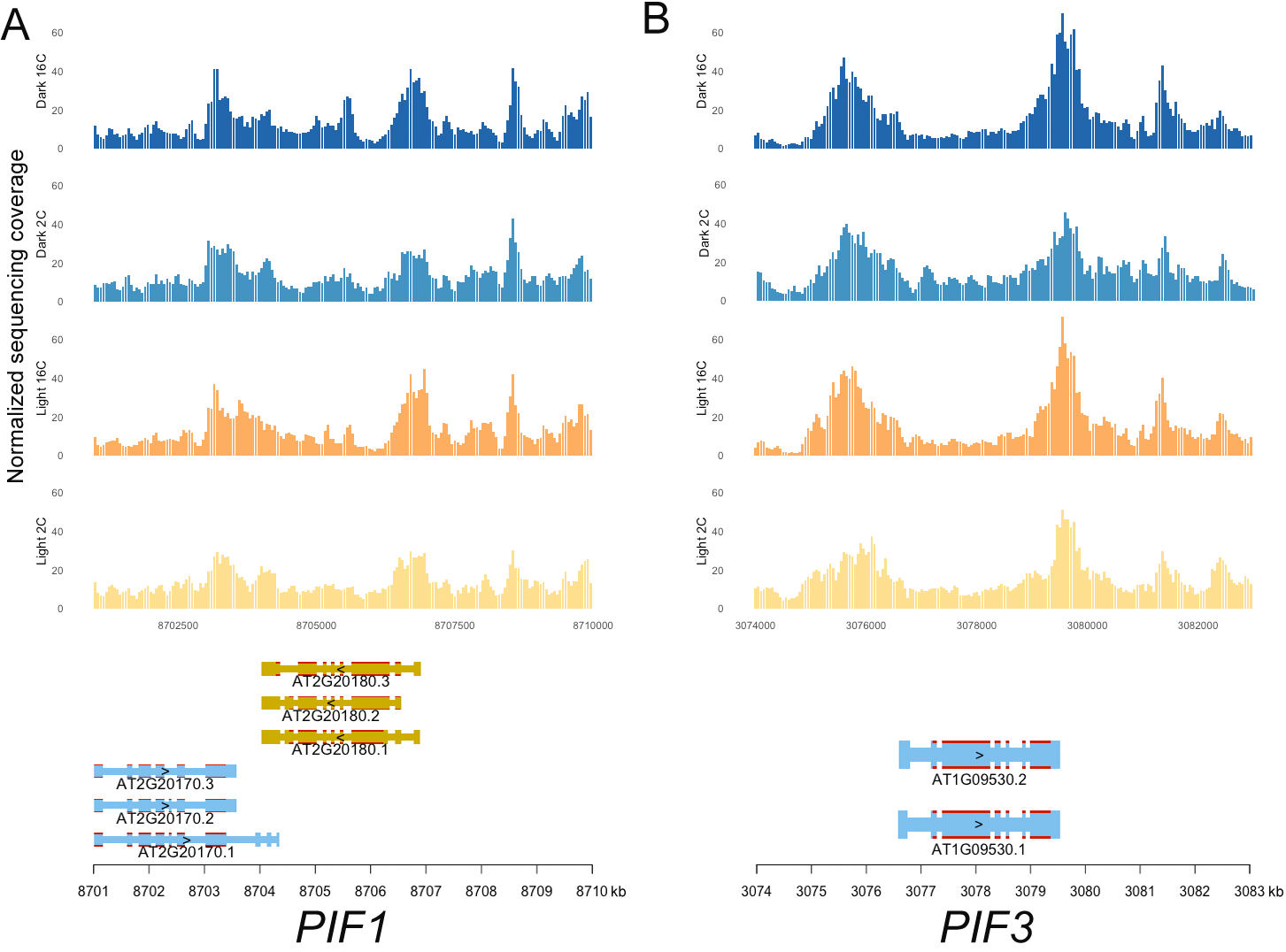

Supplement: Supplementary file 1 [file plants-09-01478-s001.zip › FigS3_v4.tif]

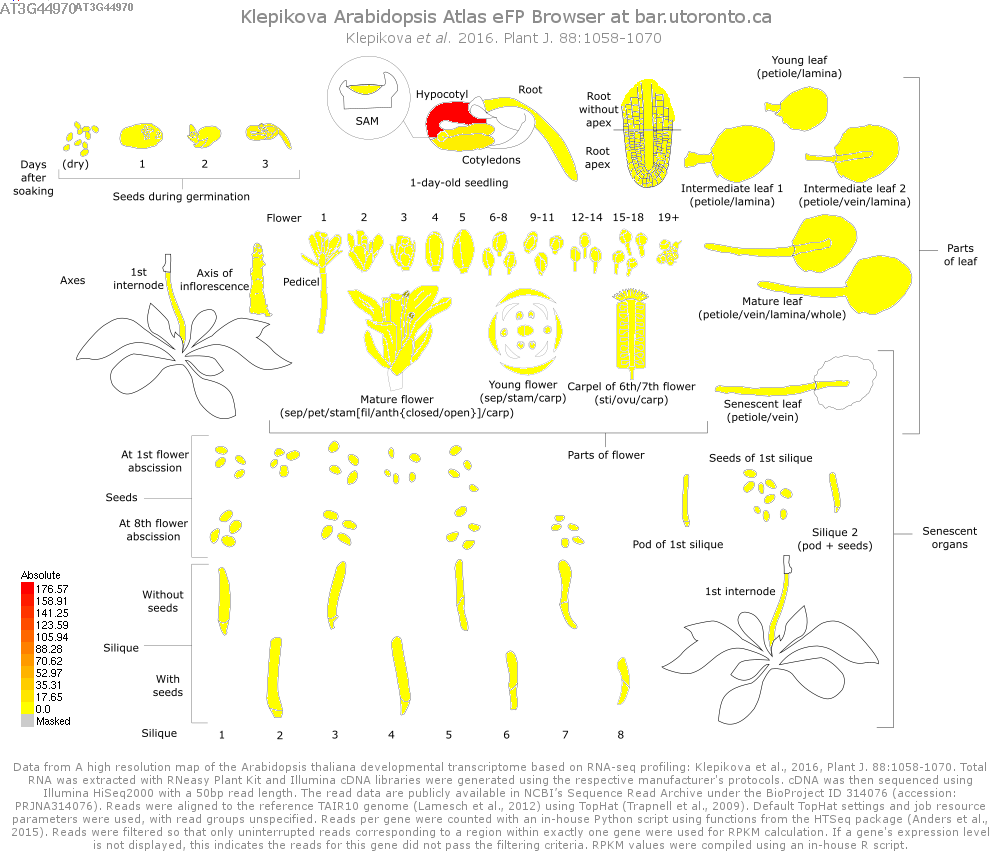

Supplement: Supplementary file 1 [file plants-09-01478-s001.zip › FigS4.png]

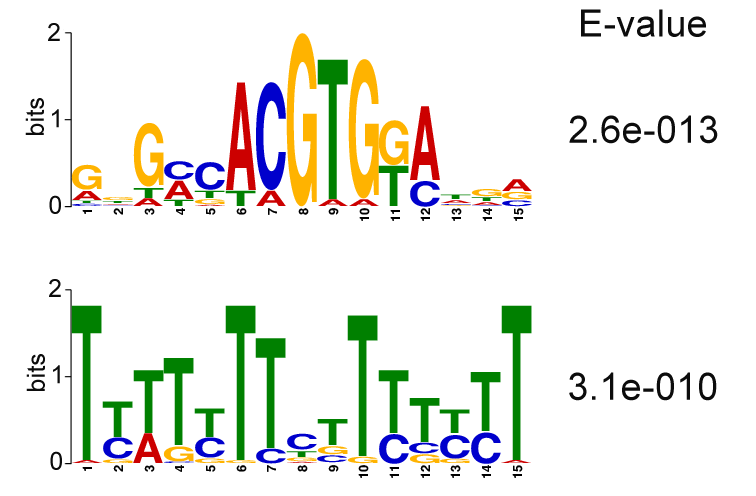

Supplement: Supplementary file 1 [file plants-09-01478-s001.zip › FigS5_v6.tif]

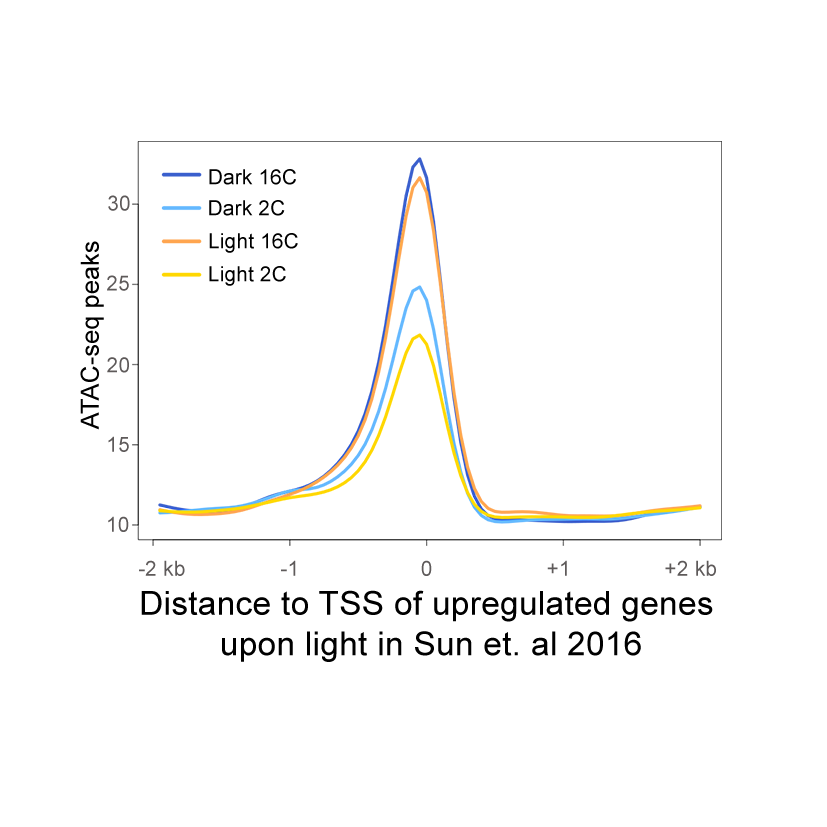

Supplement: Supplementary file 1 [file plants-09-01478-s001.zip › FigS6_v7.tif]

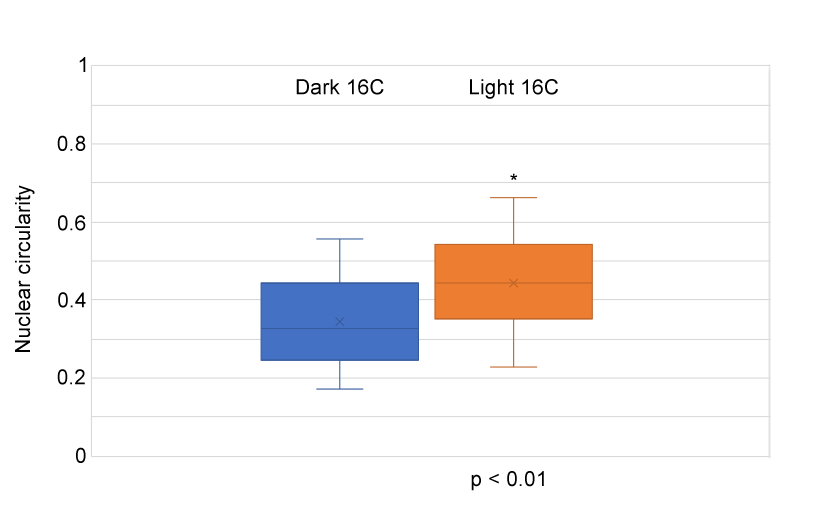

Supplement: Supplementary file 1 [file plants-09-01478-s001.zip › FigS7_v7.tif]
